# P2x7 Receptor Signaling Blockade Reduces Lung Inflammation and Necrosis During Severe Experimental Tuberculosis

**DOI:** 10.3389/fcimb.2021.672472

**Published:** 2021-05-05

**Authors:** Igor Santiago-Carvalho, Gislane de Almeida-Santos, Caio César Barbosa Bomfim, Paula Carolina de Souza, Juan Carlo Santos e Silva, Bruno Marcel Silva de Melo, Eduardo Pinheiro Amaral, Marcos Vinícios Pinheiro Cione, Elena Lasunskaia, Mario Hiroyuki Hirata, José Carlos Farias Alves-Filho, Helder Imoto Nakaya, José Maria Alvarez, Maria Regina D’Império Lima

**Affiliations:** ^1^ Departamento de Imunologia, Instituto de Ciências Biomédicas (ICB), Universidade de São Paulo (USP), São Paulo, Brazil; ^2^ Departamento de Análises Clínicas e Toxicológicas, Faculdade de Ciências Farmacêuticas (FCF), USP, São Paulo, Brazil; ^3^ Departamento de Farmacologia, Faculdade de Medicina de Ribeirão Preto, USP, São Paulo, Brazil; ^4^ Immunobiology Section, Laboratory of Parasitic Diseases, National Institute of Allergy and Infectious Diseases, National Institutes of Health, Bethesda, MD, United States; ^5^ Laboratório de Biologia do Reconhecer, Universidade Estadual do Norte Fluminense Darcy Ribeiro, Campos dos Goytacazes, Brazil

**Keywords:** tuberculosis, lung damage, adjuvant treatment, P2X7 receptor, host-direct therapies

## Abstract

The risk of developing severe forms of tuberculosis has increased by the acquired immunodeficiency syndrome (AIDS) epidemic, lack of effective drugs to eliminate latent infection and the emergence of drug-resistant mycobacterial strains. Excessive inflammatory response and tissue damage associated with severe tuberculosis contribute to poor outcome of the disease. Our previous studies using mice deficient in the ATP-gated ionotropic P2X7 receptor suggested this molecule as a promising target for host-directed therapy in severe pulmonary tuberculosis. In this study, we assessed the effects of P2X7 pharmacological blockade on disease severity. First, we observed an increase in *P2RX7* gene expression in the peripheral blood of tuberculosis patients compared to healthy donors. Lung leukocytes of mice infected with hypervirulent mycobacteria also showed increased expression of the P2X7 receptor. P2X7 blockade in mice with advanced tuberculosis recapitulated in many aspects the disease in P2X7-deficient mice. P2X7-directed therapy reduced body weight loss and the development of inflammatory and necrotic lung lesions, as well as delayed mycobacterial growth. Lower TNF-α production by lung cells and a substantial reduction in the lung GR-1^+^ myeloid cell population were observed after P2X7 inhibition. The effector CD4^+^ T cell population also decreased, but IFN-γ production by lung cells increased. The presence of a large population with characteristics of myeloid dendritic cells, as well as the increase in IL-6 production by lung cells, also indicate a qualitative improvement in the pulmonary immune response due to P2X7 inhibition. These findings support the use of drugs that target the P2X7 receptor as a therapeutic strategy to improve the outcome of pulmonary tuberculosis.

## Highlights

The P2X7 receptor detects ATP released during stress or cell death and activates the NLRP3 inflammasome, leading to mature IL-1β and IL-18 secretion and cell death by pyroptosis. Notably, prolonged stimuli of the P2X7 receptor induce necrotic cell death due to formation of large pores in the cell membrane. The P2X7 receptor has been previously suggested as a promising target candidate for host-directed therapies in severe pulmonary tuberculosis. In this study, we provide proof of concept for this approach in mice infected with hypervirulent mycobacteria. P2X7-directed therapy administered over a short period of time in mice with advanced pulmonary tuberculosis was effective in reducing disease severity. This therapeutic strategy can be particularly useful, combined with anti-microbial drugs, to interrupt the vicious cycle of uncontrolled inflammatory response and damage to lung tissue in severe forms of the disease.

## Introduction

Tuberculosis (TB) is an airborne infectious disease that remains as one of the major causes of health threat (World Health Organization, 2020). In 2019, the estimated number of fatal cases resulting from *Mycobacterium tuberculosis* infection reached 1.4 million worldwide, making TB one of the top ten causes of global death (World Health Organization, 2020). Multiple immune evasion strategies developed along the coevolution of mycobacteria with the vertebrate host, such as the interference with antigen presentation by major histocompatibility complex (MHC) class II molecules, explain the limited success of vaccines against TB (Ernst, 2018). TB control is also hampered by the acquired immunodeficiency syndrome (AIDS) epidemic, lack of effective drugs to eliminate latent infection as well as the emergence of drug-resistant mycobacterial strains (Shah et al., 2007; World Health Organization, 2020). Failure to prevent and control *M. tuberculosis* infection increases the risk of developing severe forms of TB (Caws et al., 2008; Bell and Noursadeghi, 2018). Severe disease is commonly associated with exacerbated lung inflammation and necrosis, resulting in serious sequelae for TB patients.

This emerging scenario has encouraged the combined use of standard anti-microbial treatments for TB with host-directed therapies based on anti-inflammatory interventions. This therapeutic approach directly targets the inflammatory response triggered by the infection to prevent and repair tissue damage, promote pathogen elimination and reduce disease sequelae (Kaufmann et al., 2014; Zumla et al., 2016; Tsenova and Singhal, 2020). Accelerating the patient healing and reducing the adverse effects of anti-microbial drugs are both desirable outcomes of adjunctive therapies (Hawn et al., 2013; Tobin, 2015). Current clinical experience of anti-inflammatory therapy in TB is mostly with corticosteroids, which have been successfully used to treat tuberculous meningitis and pericarditis, as well as to ameliorate paradoxical HIV-TB immune reconstitution inflammatory syndrome (Prasad et al., 2016; Kaufmann et al., 2018; Schutz et al., 2018). In pulmonary TB, the benefits of corticosteroid treatment are limited to clinical parameters, such as fever reduction and weight gain (Bilaçeroğlu et al., 1999). Thus, it would be of great interest to establish new therapeutic approaches to protect lung tissue from the harmful effects of uncontrolled inflammation caused by *M. tuberculosis* infection.

Our previous studies have highlighted the extracellular ATP sensing by the P2X7 receptor cation channel as a promising target candidate for host-directed therapies in severe pulmonary TB (Amaral et al., 2014; Bomfim et al., 2017). Although a protective role in extra pulmonary TB has been attributed to the P2X7 receptor (Fernando et al., 2007), its effect on patients with severe pulmonary disease is unknown. ATP released at high concentrations during cell stress or death acts as a damage signal and activates the P2X7 receptor. The influx of Ca^2+^ and efflux of K^+^ cause cytoplasmic ionic changes, which lead to NLRP3-inflammasome activation and culminates in mature IL-1β and IL-18 secretion and cell death through pyroptosis (Mariathasan et al., 2006; Iyer et al., 2009). Importantly, prolonged stimuli of the P2X7 receptor induce necrotic cell death due to formation of large membrane pores (Di Virgilio et al., 1989; Di Virgilio et al., 2017). P2X7 deficiency, particularly in bone marrow-derived cells, improves lung disease in mice infected with hypervirulent mycobacteria by reducing the inflammatory response, necrotic lesions and bacterial load (Amaral et al., 2014; Bomfim et al., 2017). P2X7 signaling aggravates lung disease by promoting the lysis of infected macrophages, facilitating bacterial release in the extracellular milieu (Amaral et al., 2014; Bomfim et al., 2017). In addition, the accumulation of myeloid-derived suppressor cells in the lungs has been shown to be dependent on P2X7 activation (Bomfim et al., 2017). This population of immature myeloid cells migrates from the bone marrow to the lungs when the disease gets worse and becomes a permissive niche for the replication of the bacillus, allowing the spread of the infection in the lungs (Knaul et al., 2014; Tsiganov et al., 2014; Lovewell et al., 2020; Barbosa Bomfim et al., 2021).

In this study, we evaluated the effects of pharmacological blockade of the P2X7 receptor on severe pulmonary TB. First, the *P2RX7* gene expression was assessed in the peripheral blood of TB patients using a public transcriptome database. The presence of the P2X7 receptor in lung leukocytes was evaluated in C57BL/6 mice infected with hypervirulent mycobacteria. The effects of P2X7 blockade in the advanced stage of the disease were then investigated in this experimental model of severe pulmonary TB. Our findings demonstrate that the *P2RX7* gene and P2X7 protein are highly expressed in human and murine TB, respectively. P2X7 inhibition prevents disease progression and is a promising approach to be used as a host-directed therapy for severe forms of pulmonary TB.

## Materials and Methods

### Transcriptome Analysis of Human Peripheral Blood

Human transcriptome data were analyzed using R. Raw data downloaded using *GEOquery* (Davis and Meltzer, 2007) obtained from the GEO datasets (GEO accession number: GSE54992). Two classes of samples were used: healthy donors as control (*N* = 6) and active TB (*N* = 9). Array quality control was applied using *arrayQualitymetrics* (Kauffmann et al., 2009) to identify outliers. Expression data were normalized using RMA function from the *affy* (Gautier et al., 2004). Probes matching for the same gene were collapsed by taking the highest expression across the samples. Differential expression analyses were performed using *limma* (Ritchie et al., 2015). The differentially expressed genes (DEGs) were plotted with log_2_ fold-change and the -log_10_ *P* adjusted value. The package *ComplexHeatmap* (Gu et al., 2016) was used to plot the expression patterns.

### Mice

Specific pathogen-free C57BL/6 male (6-8-week-old) mice were bred at the isogenic mouse facility, ICB, USP. After infection, mice were maintained in micro isolator cages with *ad libitum* feed at the Biosafety Level 3 facility, FCF, USP. All procedures were performed in accordance with national regulations of the ethical guidelines for mouse experimentation with permit number 5611150818 and 136/2017.

### Mycobacterial Culture and Mouse Infection

The frozen bacilli were thawed and grow in Middlebrook 7H9 medium enriched with 10% (vol/vol) ADC (albumin, dextrose, catalase) (Difco, BD Biosciences, USA), 0.4% (mass/vol) sodium pyruvate (Sigma-Aldrich, USA) and 0.05% (vol/vol) Tween 80 (Sigma-Aldrich), and maintained at 37°C for 7 days until mid-log phase (OD 0.6 – 0.9). Bacterial concentration was determined using a spectrophotometer at 600 nm. Mice were anesthetized intraperitoneally (i.p.) with ketamine (Vetbrands, Brazil; 100 mg/kg) and xylazine (Vetbrands; 15 mg/kg) and infected intratracheally (i.t.) with ~100 bacilli of the *Mycobacterium bovis* MP287/03 strain (Amaral et al., 2014).

### Brilliant Blue G Treatment

For *in vivo* pharmacological blockade of the P2X7 receptor, mice were injected i.p. every 2 days with brilliant blue G (BBG, Sigma-Aldrich) (45 mg/Kg/mouse in 300 µL of PBS), starting on day 21 of infection.

### Lung Macroscopic and Microscopic Analyses

The harvested lung lobes were washed with sterile PBS and weighed. The lung relative mass was calculated by dividing the mean of lung weight in experimental mice by the mean of lung weight in uninfected controls. The right lung upper lobe was maintained in 10% buffer formalin, photographed and subsequently embedded in paraffin. Histological sections of approximately 4-5 μm were stained using the hematoxylin-eosin (HE) method for tissue morphological analysis and the Ziehl Neelsen (ZN) method for mycobacterial visualization. The microscopic analyses were performed with a Leica microscope (Germany), and images were captured with a Nikon camera (Japan).

### Lung Cell Harvesting and Counting

The lung lobes were dissected and digested with collagenase type IV (0.5 mg/mL, Sigma-Aldrich) in RPMI 1640 medium (Gibco, USA) at 37°C for 40 minutes under agitation (200 rpm) (Amaral et al., 2019b). The lung cells were dissociated by passage through a 100 µm pore-size cell strainer and incubated with ACK Lysing Buffer (Thermo Fisher Scientific, USA) at room temperature for one minute to deplete the erythrocytes. The lung cell suspensions were washed with 10% fetal calf serum (FCS, Gibco) in PBS following centrifugation at 1,200 rpm for 5 minutes and resuspended in RPMI 1640 medium enriched with 10% FCS and 0.1% gentamicin (Gibco). The viable lung cell numbers were determined using trypan blue exclusion assay and a hemocytometer.

### Flow Cytometry Analysis

Lung cells (1×10^6^ cells/well) were seeded in round-bottom 96-well plates and stained using fluorochrome-labeled monoclonal antibodies to CD45 (30-F11), CD11b (M1/70), CD11c (N418), GR1 (RB6-8C5), CD4 (RM4.5), CD44 (IM7), CD69 (H1.2F3), P2X7 (1F11), lineage (CD4- RM4-5; CD8-S3-6.7; CD19 - 1D3 and NK.1 - PK136 (BD Biosciences). Live/dead dye (Thermo Fisher Scientific) was used to stain dead cells, as described in data sheet. Cells were fixed with 4% paraformaldehyde and analyzed with the LSRFortessa™ flow cytometer (BD Bioscences – USA) and the FlowJo 10.4.2 software (BD Biosciences). The gate strategy for analysis of CD11b^+^ myeloid cells and CD4^+^ T cells are shown in the **Supplementary Figures 1A, B**.

### Colony-Forming Unit (CFU) Counting

Serial dilutions of lung homogenates were cultured in 6-well plates with Middlebrook 7H10 Agar supplemented with 10% (vol/vol) OADC (oleate, albumin, dextrose and catalase) (Difco, BD Biosciences) and 0.4% (mass/vol) sodium pyruvate, at 37°C for 21 days. CFUs were counted visually.

### Cytokine Quantification

Cells (1×10^6^ cells/well) harvested from the lungs were cultured in sterile round-bottom 96-well plates in complete RPMI 1640 medium enriched with 10% FCS, 2 mM glutamine, 1 mM sodium pyruvate and 0.05% gentamicin for 48 hours at 37°C and 5% CO_2._ The supernatants were collected, filtered and the concentrations of TNF-α, IL-6, IL-10 and IFN-γ cytokines were determined using the appropriated Mouse ELISA kit, as described in data sheet (BD OptEIA, USA).

### Statistical Analyses

Statistical analyzes were performed using the GraphPad Prism 6 software. Data were described as mean ± standard error. The Mann-Whitney non-parametric T test was used to assess differences between two groups. The one-way ANOVA and Tukey’s *post hoc* tests were used to compare three or more groups. Differences between groups were considered significant when *p* < 0.05.

## Results

### Increased Expression of the *P2RX7* Gene in the Peripheral Blood of TB Patients and the P2X7 Receptor on Lung Leukocytes of Mice With Severe TB

**Figure 1:**
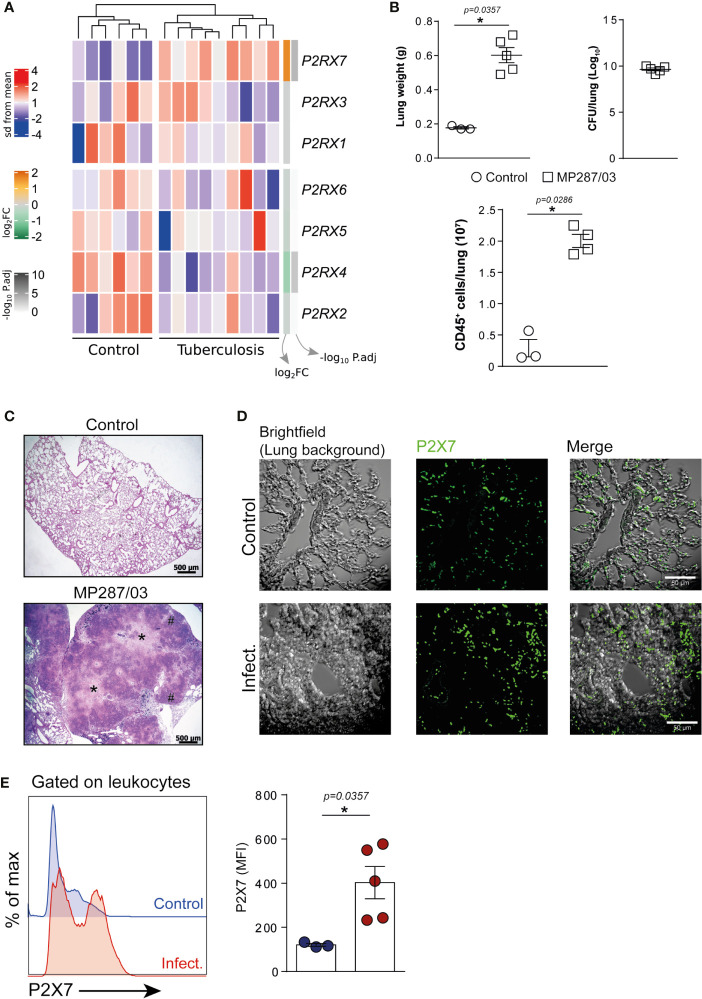
The expression of *P2RX7* gene and P2X7 receptor is increased in the peripheral blood of TB patients and on lung leukocytes of mice with severe TB. **(A)** Gene expression counts were z-score normalized across all samples. Log_2_ fold-change and -log_10_ P. adjust from DEG analysis are shown in the right annotation of the heat map. Genes were ordered by hierarchical clustering (Euclidean distance). **(B–E)** C57BL/6 mice were infected i.t. with ~100 MP287/03 bacilli. Uninfected mice were used as control group. Mouse lungs were evaluated at day 28 of infection. **(B)** Lung weights, CFUs per lung and leukocyte (CD45^+^) cell numbers per lung are shown. **(C)** Representative lung sections stained with hematoxylin-eosin method (scale bars correspond to 500 µm) of infected and control group. Asterisks (*) indicate necrotic areas and hash signs (#) indicate alveolitis. **(D)** Immunofluorescence staining for the P2X7 receptor (green) in representative lung sections (scale bars correspond to 50 µm). **(E)** Histograms show P2X7 expression in lung leukocytes. Mean fluorescence intensities (MFIs) of P2X7 expression are shown in the bar graph. Significant differences were observed between indicated groups with *p < 0.05, using Mann-Whitney non-parametric T test. Data are representative of two independent experiments with three to five mice in each group.

To investigate whether the *P2RX7* gene was expressed differently in TB patients, we re-analyzed the peripheral blood transcriptome data from healthy individuals and patients with the active disease (Cai et al., 2014). Among the P2X family members, only the *P2RX7* gene was upregulated in TB patients when compared to healthy individuals (mean log_2_ fold-change = 1.58, adjusted *P* value = 1.81e-04) (**Figure 1A**). Next, we assessed the expression of the P2X7 receptor on lung cells isolated from C57BL/6 mice infected i.t. with ~100 *M. bovis* bacilli of the hypervirulent MP287/03 strain. This experimental model proved to be useful to understand the role of P2X7 receptor in the development of severe forms of pulmonary TB (Amaral et al., 2014; Bomfim et al., 2017). As previously reported, C57BL/6 mice developed severe pneumonia characterized at day 28 p.i. by increased lung weight, high bacterial load and intense leukocyte infiltration (**Figure 1B**), as well as extensive areas of intra-granulomatous necrosis (**Figure 1C**). Immunofluorescence analysis of lung tissue revealed many cells expressing the P2X7 receptor in infected and uninfected mice; the expression level was apparently higher in infected mice (**Figure 1D**). P2X7 upregulation was confirmed by flow cytometry analysis, showing higher P2X7 expression on lung leukocytes isolated from infected mice compared to uninfected mice (**Figure 1E**). Increased P2X7 expression can make immune cells highly responsive to extracellular ATP, as previously reported in experimental models of autoimmune disease and malaria (Proietti et al., 2014; Salles et al., 2017).

### Protective Effects of P2X7 Pharmacological Blockade on the Development of Severe Pulmonary TB in Mice

**Figure 2:**
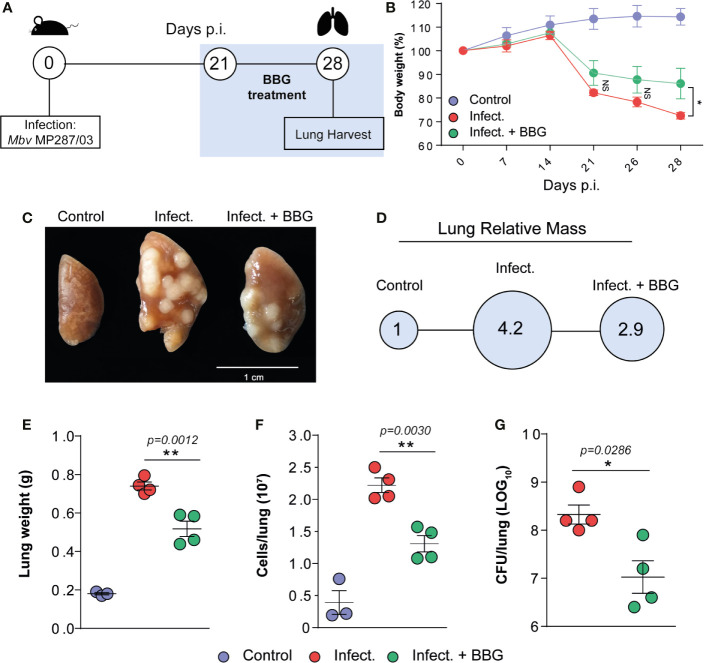
P2X7 pharmacological blockade at advanced pulmonary TB reduces the disease severity in mice. C57BL/6 mice were infected i.t. with ~100 MP287/03 bacilli. Uninfected mice were used as control group. The P2X7 inhibitor BBG was administered i.p. at a dose of 45 mg/Kg, every 2 days, from day 21 of infection. Infected (untreated) group received the vehicle (PBS). Mouse lungs were evaluated at day 28 of infection. **(A)** Schematic representation of the experimental BBG treatment protocol is shown. **(B)** Mouse body weights were determined weekly. **(C, D)** Macroscopic images of representative lung lobes and relative lung masses (circles) are shown. **(E–G)** Lung weights, cell numbers per lung and CFUs per lung are shown. Significant differences were observed between the indicated groups with *p < 0.05 and **p < 0.01, using One-way ANOVA and Tukey’s *post hoc* tests or Mann-Whitney non-parametric T test. The statistical differences between uninfected and infected groups are not shown. Data are representative of two independent experiments with three to four mice in each group.

To evaluate the effects of P2X7 pharmacological blockade during advanced pulmonary TB, C57BL/6 mice infected i.t. with MP287/03 mycobacteria and uninfected mice were treated i.p. with the P2X7 antagonist BBG (**Figure 2A** and **Supplementary Figure 2A**). BBG is a food additive with structure and function analogous to highly selective P2X7 antagonists, which was first used to improve tissue recovery after spinal cord injury in rats (Peng et al., 2009). P2X7-directed therapy started on day 21 p.i. when a reduction of more than 10% of body weight indicated the advanced stage of the disease (**Figure 2B**). Notably, P2X7 inhibition prevented body weight loss until day 28 of infection. Fewer lung white nodes and reduced lung relative masses were observed in BBG-treated mice compared to untreated animals (**Figures 2C, D**). Lung weight, cellularity and bacterial burden were also lower in BBG-treated mice (**Figures 2E–G**). In contrast, BBG treatment had no effect on lung weight and cellularity in uninfected mice (**Supplementary Figures 2B, C**).

**Figure 3:**
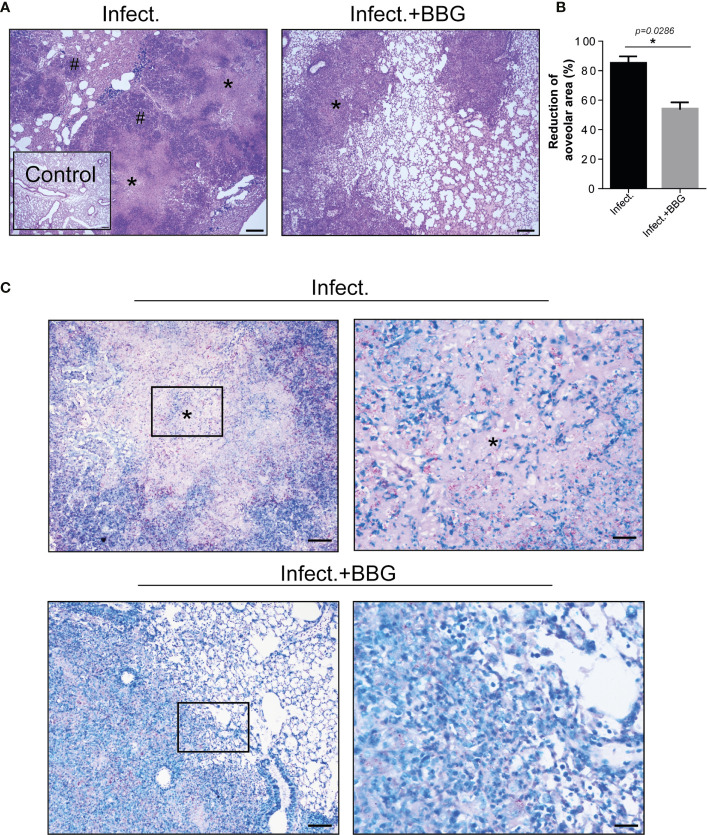
P2X7 pharmacological blockade reduces tuberculous pulmonary lesions in mice. Histopathological changes in lungs of the BBG-treated and untreated mice, previously infected with ~100 MP287/03 bacilli, were evaluated at day 28 of infection. **(A)** Images of representative lung sections stained with hematoxylin-eosin method (scale bars correspond to 200 µm) are shown. Asterisks (*) indicate necrotic areas and hash signs (#) indicate alveolitis. **(B)** Morphometric quantification of aerated alveolar space is shown. **(C)** Images of representative lung sections stained with Ziehl Neelsen method. Magnified areas from left squares are demonstrated in right images (scale bars correspond to 100 µm and 25 µm, respectively), showing large number of extracellular bacilli in necrotic regions (*) in untreated mice, whereas predominantly intracellular bacilli were seen in the BBG-treated mice. Significant differences were observed between indicated groups with *p < 0.05, using Mann-Whitney non-parametric T test. Data are representative of two independent experiments with four mice in each group.

Histopathological analysis on day 28 p.i. revealed better preserved lung tissue in BBG-treated mice compared to untreated controls (**Figure 3A**), which was corroborated by morphometric quantification of the aerated alveolar space (**Figure 3B**). Areas of alveolitis and necrosis were substantially reduced after P2X7 inhibition (**Figure 3A**). In addition, extracellular bacilli were found in abundance in necrotic lesions in untreated mice, but not in BBG-treated mice, where solid granulomas with predominantly intracellular bacilli were seen (**Figure 3C**). Together, these findings demonstrate that P2X7 pharmacological blockade prevents the development of severe forms of pulmonary TB pathology in mice.

### Reduced Leukocyte Recruitment in Mice With Advanced TB Treated With P2X7-Directed Therapy

**Figure 4:**
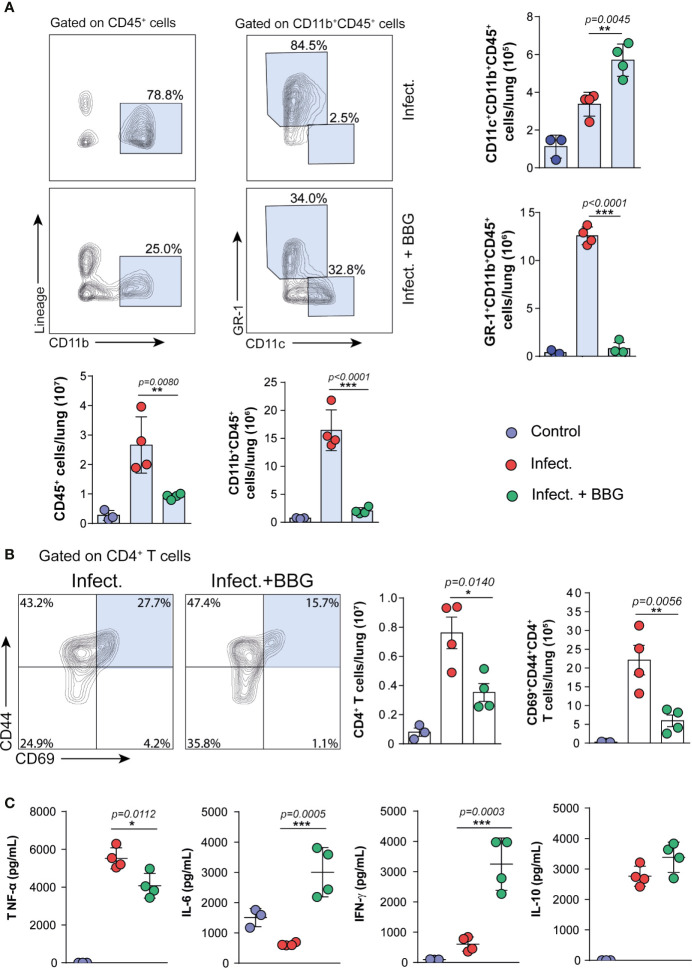
P2X7-directed therapy reduces the pulmonary inflammatory response, but increases the population of CD11c^+^ myeloid cells and the production of IFN-γ and IL-6 by lung cells. Lung cells from BBG-treated and untreated mice were evaluated at day 28 p.i. with ~100 MP287/03 bacilli. Lung cells from uninfected mice were used as control. **(A)** Contour-plots show the expression of CD11b *vs* lineage in CD45^+^ cells and CD11c *vs* GR-1 in CD11b^+^CD45^+^ cells. Bar graphs show cell numbers per lung. **(B)** Contour-plots show CD44 and CD69 expression in CD4^+^ T cells. Bar graphs show cell numbers per lung. **(C)** Cytokine levels in 48h-culture supernatants of lung cells are shown. Significant differences were observed between the indicated groups with *p < 0.05, **p < 0.01 and ***p < 0.001, using One-way ANOVA and Tukey’s *post hoc* tests. The statistical differences between uninfected and infected groups are not shown. Data are representative of two independent experiments with three to four mice in each group.

The pulmonary immune response was then assessed in C57BL/6 mice infected with MP287/03 mycobacteria and treated with BBG. P2X7-directed therapy caused a substantial reduction in the recruitment of leukocyte (CD45^+^) population to the lungs (**Figure 4A**). Among CD11b^+^ myeloid cells, the GR1^+^ population was particularly diminished after P2X7 blockade. Notably, the population of myeloid cells expressing CD11c was increased in BBG-treated mice compared to untreated animals. Regarding CD4^+^ T cells, P2X7-directed therapy impaired the accumulation of total and CD69^+^CD44^+^ populations (**Figure 4B**). This phenotype is characteristic of effector CD4^+^ T cells that infiltrate the pulmonary parenchyma in mice infected with mycobacteria (Sakai et al., 2014). In the absence of infection, similar numbers of these cell populations were observed in BBG-treated and untreated mice (**Supplementary Figures 2D, E**). In addition, a lower concentration of TNF-α was found in lung cell supernatants from infected mice treated with BBG compared to those not treated (**Figure 4C**). Remarkably, low levels of IFN-γ and IL-6 were secreted by lung cells from infected mice; BBG treatment increased substantially the production of these cytokines. IL-10 was produced at similar levels by lung cells from both infected mouse groups. Comparable baseline levels of TNF-α, IL-6, IFN-γ and IL-10 were found in lung cell supernatants from uninfected mice, treated or not with BBG (**Supplementary Figure 2F**).

**Figure 5:**
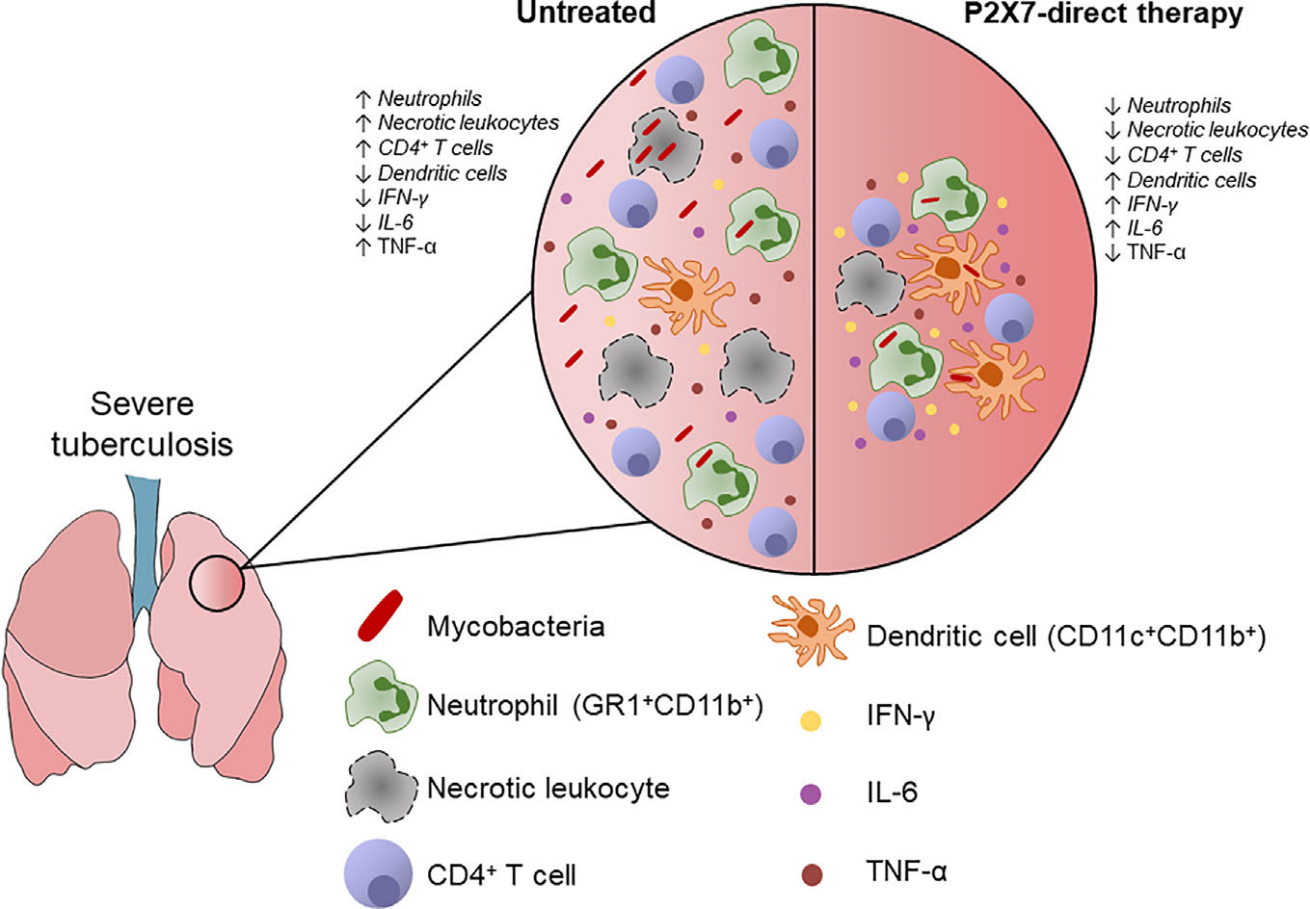
Schematic illustration shows the effects of P2X7-directed therapy on the pulmonary inflammatory response to hypervirulent mycobacterial infection. P2X7-mediated necrotic death of lung leukocytes promote bacillus spread and ATP release to the extracellular milieu. Extracellular ATP at high concentration stimulates the P2X7 receptor and exacerbates inflammatory and necrotic lung lesions. The P2X7-direct therapy reduces the recruitment of lung leukocytes, particularly neutrophils (GR1^+^CD11b^+^), as well as the production of TNF-α. The effector CD4^+^ T cell population was also decreased, but IFN-γ production by lung cells was increased. P2X7 inhibition also increases the population of myeloid dendritic cells (CD11c^+^CD11b^+^) and the production of IL-6 by lung cells, indicating a qualitative improvement in the pulmonary immune response. This scenario supports the use of therapeutic interventions in the P2X7 receptor to increase the effectiveness of anti-microbial treatment and reduce the severity of pulmonary TB.

In resume, P2X7-directed therapy during advanced TB impairs the recruitment of GR1^+^ myeloid cells and CD4^+^ T cells to the lungs (**Figure 5**). The increase in a myeloid cell population with characteristics of dendritic cells, as well as in production of IFN-γ and IL-6 by lung cells, suggests a qualitative improvement in the pulmonary immune response due to P2X7 inhibition.

## Discussion

This study supports the use of drugs that target the P2X7 receptor as a therapeutic strategy to improve the outcome of pulmonary TB. This approach can be particularly useful, in combination with anti-microbial drugs, to interrupt the vicious cycle of uncontrolled inflammatory response and damage to lung tissue in severe forms of the disease.

Among P2X (1-7) family members, the *P2RX7* gene expression was the only one increased in peripheral blood of TB patients in relation to healthy donors. Another indication that P2X7 signaling is a promising target for host-directed therapies in pulmonary TB was the increased expression of this receptor in lung leukocytes of mice infected with hypervirulent mycobacteria. Extracellular ATP at high concentration in severe TB pneumonia could lead to strong activation of the NLRP3 inflammasome in leukocytes expressing high levels of the P2X7 receptor. The release of large amounts of pro-inflammatory cytokines and the pyroptotic death of numerous immune cells would be expected in this scenario. In addition, both gasdermin D cleaved by caspase-1 and pannexin-1 activated by the influx of Ca^2+^ form pores in the cell membrane, allowing ATP release in the extracellular environment (Locovei et al., 2006; Shi et al., 2015; Sborgi et al., 2016). Accumulation of extracellular ATP may boost the inflammatory response and promote tissue damage. Extracellular ATP in millimolar concentrations induces pore formation and necrotic cell death through P2X7 activation (Di Virgilio et al., 1989; Di Virgilio et al., 2017). By inhibiting the P2X7 signaling pathway these processes can be interrupted.

P2X7 pharmacological blockade in mice with advanced pulmonary TB recapitulated in many aspects the disease in P2X7-deficient mice infected with hypervirulent mycobacteria (Amaral et al., 2014). P2X7-directed therapy administered over a short period of time was effective in reducing body weight loss and the development of inflammatory and necrotic lung lesions, as well as delaying mycobacterial growth. The reduction in body weight loss in infected mice treated with P2X7 inhibitor may result from the lower production of TNF-α by lung leukocytes, as this cytokine was originally identified by its ability to induce cachexia (Cerami and Beutler, 1988). The decrease in pulmonary necrotic lesions may be due to the inhibition of necrotic cell death, as a consequence of P2X7 signaling blockade. Specifically, *P2RX7* ^-/-^ macrophages infected with hypervirulent mycobacteria are more resistant to cell death induced by high levels of extracellular ATP and release fewer bacteria to the extracellular milieu than wild-type macrophages (Amaral et al., 2014). Supporting this *in vitro* finding, fewer extracellular bacilli were found in the lung tissue when the P2X7 receptor was inhibited *in vivo*.

The reduction in tissue damage and bacterial load, leading to less stimulation of the immune system by damage and pathogen-associated molecular patterns, may explain the limited areas of lung inflammation in infected mice given the P2X7-directed therapy. A similar approach in which ferroptosis was inhibited in mice acutely infected with *M. tuberculosis* also reduces lung inflammatory lesions and mycobacterial burden (Amaral et al., 2019a). P2X7 signaling blockade may reduce the release of damage signals, impairing the activation of macrophages and, consequently, the secretion of pro-inflammatory cytokines, such as TNF-α. P2X7 inhibition may also restrain the activation of NLRP3 inflammasome and the release of mature IL-1β and IL-18, promoting control of the inflammatory response. However, on day 28 p.i. with MP287/03 mycobacteria, IL-1β is produced at low levels by lung cells from both C57BL/6 and P2X7-deficient mice (Amaral et al., 2014), suggesting a minor role for this cytokine in this TB model. A direct effect of P2X7 inhibition on Ca^2+^ influx may also have impaired leukocyte activation and contributed to restrict the inflammatory response. Supporting this idea, P2X7 signaling in myeloid cells induces the expression of several chemokines that promote leukocyte recruitment, such as monocyte chemoattractant protein 1 (MCP-1, CCL2), IL-8, CC-ligand 3 (CCL3) and CXC-ligand 2 (CXCL2), as well as the production of pro-inflammatory cytokines, such as TNF-α (Shieh et al., 2014; Di Virgilio et al., 2017). A reduction in TNF-α production due to P2X7 blockade may also have contributed to restrain necrotic lung lesions, as excessive production of this cytokine can result in the development of tissue-damaging immunopathology (Dorhoi and Kaufmann, 2014).

The population of GR-1^+^ myeloid cells was particularly affected by P2X7 pharmacological blockade. Characterized as granulocytic myeloid-derived suppressor cells, GR-1^+^ cells accumulate massively in the lungs during the final stage of hypervirulent mycobacterial infection, promoting bacterial growth and the development of necrotizing pneumonia (Barbosa Bomfim et al., 2021). This immature myeloid cell population is generated by emergency hematopoiesis in response to excessive or chronic infections (Boettcher and Manz, 2017). Therefore, the reduction in the GR-1^+^ cell population demonstrates the potential of P2X7-directed therapy to help healing severe pulmonary TB. The effector CD4^+^ T cell population also decreased due to P2X7 inhibition. Interestingly, elevated production of IFN-γ was found *ex vivo* in lung cell suspension of mice receiving BBG, suggesting an improvement in the effector response of these cells and/or an increase in IFN-γ production by other cell subsets.

Adenosine, presumably generated through ATP degradation by ectonucleotidases, has been implicated in the suppression of IFN-γ production by lung CD4^+^ T cells in this experimental model of severe TB. The pharmacological inhibition of adenosine receptors increased the frequency of IFN-γ-producing CD4^+^ T cells (Amaral et al., 2019b), which are the major source of IFN-γ in the lungs of MP287/03-infected mice at day 28 p.i. (Barbosa Bomfim et al., 2021). The protection of infected lung tissue resulting from P2X7 inhibition may prevent ATP release and, consequently, the accumulation of adenosine in the extracellular environment, leading to an increase in IFN-γ production by CD4^+^ T cells. IFN-γ production has a crucial role in resistance to *M. tuberculosis* infection (Flynn et al., 1993), and may have contributed to control the lung bacterial burden in infected mice treated with P2X7-directed therapy. The presence of a large population with characteristics of myeloid dendritic cells in the mouse lungs (Misharin et al., 2013), as well as the increased *ex vivo* production of IL-6 by lung cells, may also indicate a qualitative improvement in the pulmonary immune response due to P2X7 inhibition. Our interpretation of these findings is that the preservation of lung tissue due to P2X7 blockade in infected mice allowed the recruitment of dendritic cells from the blood to the lungs. This process occurs constantly by a steady-state bone marrow output and is rapidly intensified by pathogenic stimuli (Holt and Schon-Hegrad, 1987; McWilliam et al., 1994). Dendritic cell migration to the lungs may have been interrupted during emergency hematopoiesis in infected and untreated mice. Like macrophages, pulmonary dendritic cells produce IL-6 after stimulation (Demedts et al., 2005), and are presumably an important source of this cytokine in infected mice given P2X7-directed therapy.

Although the P2X7 receptor has been suggested previously as a promising target candidate for therapies in severe pulmonary TB (Amaral et al., 2014), our present findings provide proof of concept for this approach in mice infected with hypervirulent mycobacteria. The P2X7-directed therapy has the particularity of intervening directly to maintain the integrity of the infected lungs, avoiding the uncontrolled inflammatory response induced by extensive tissue damage. In addition, therapeutic intervention in the P2X7 receptor seems to improve the quality of the immune response to severe mycobacterial infection. A possible adverse effect of P2X7 inhibition would be the increase in mycobacterial resistance to anti-TB treatment, resulting from the limited effect of antibiotics on bacilli located in solid granulomas. However, this host-directed therapy can be decisive for a favorable outcome in severe cases if administered in conjunction with anti-TB drugs with ability to eradicate persistent bacilli, such as pyrazinamide (Whitfield et al., 2015). This therapeutic strategy can be exploited to increase the effectiveness of anti-TB treatment in severe pulmonary TB.

## Data Availability Statement

The original contributions presented in the study are included in the article/**Supplementary Material**. Further inquiries can be directed to the corresponding authors.

## Ethics Statement

The animal study was reviewed and approved by Animal Care Committee of Institute of Biomedical Science with permit number 5611150818 and 136/2017.

## Author Contributions 

Conceived and designed the experiments: IS-C; GA-S; CCBB; HI and MRDL. Performed the experiments: IS-C; GA-S; CCBB; PCS; BS and MP. Analyzed the data: IS-C; GA-S; CCBB; JCSS and MRDL. Contributed reagents/materials/analysis tools: IS-C; GA-S; CB; EPA; MHH; EL; JCFA-F; HIN; JMA and MRDL.

## Funding

This study was supported in whole by São Paulo Research Foundation (FAPESP-Brazil) grants: 2015/20432-8 (MRDL), 2019/24700-8 (IS-C), 2017/11030-9 (PCS), 2019/27139-5 (JCSS) and 2020/09043-8 (CCBB); and by National Council for Scientific and Technological Development (CNPq) grants: 408909/2018-8 (MRDL), 303810/2018-1 (MRDL) and 140666/2018-4 (GA-S). All authors contributed to the article and approved the submitted version.

## Conflict of Interest

The authors declare that the research was conducted in the absence of any commercial or financial relationships that could be construed as a potential conflict of interest.

## Supplementary Material

Supplementary Figure 1 Gate strategies for the analyses of myeloid cells and CD4^+^ T cells are shown in mice infected with MP287/03 mycobacteria, treated or not treated with BBG. C57BL/6 mice were infected i.t. with ~100 MP287/03 bacilli. Lung cells were evaluated at day 28 of infection. **(A)** Gate strategies to evaluate CD45^+^, CD45^+^Lin^-^CD11b^+^, CD45^+^Lin^-^CD11b^+^CD11c^+^, CD45^+^Lin^-^CD11b^+^GR1^+^ cells are shown. **(B)** Gate strategies to evaluate CD4^+^ T cells and CD69^+^CD44^+^CD4^+^ T cells are shown.

Supplementary Figure 2 BBG treatment does not interfere with pulmonary immunological parameters in uninfected C57BL/6 mice. Four doses of BBG (45 mg/kg) were administered i.p. every two days in C57BL/6 mice, and lung cells were harvested 7 days after the first dose. Untreated C57BL/6 mice were used as controls. **(A)** Schematic representation of the experimental BBG treatment protocol is shown. **(B)** Lung weights are shown. **(C)** Cell numbers per lung are shown. **(D)** CD45^+^, CD11b^+^CD45^+^ and CD11c^+^CD11b^+^CD45^+^, GR1^+^CD11b^+^CD45^+^ cell numbers per lung are shown. **(E)** CD4^+^ and CD69^+^CD44^+^CD4^+^ T cell numbers per lung are shown. **(F)** Cytokine levels in 48h-culture supernatants of lung cells are shown. No significant difference was observed using Mann-Whitney non-parametric T test.
