# Supplementary figures and images for: P2x7 Receptor Signaling Blockade Reduces Lung Inflammation and Necrosis During Severe Experimental Tuberculosis
Source: Front Cell Infect Microbiol. 2021 May 5;11:672472. doi: 10.3389/fcimb.2021.672472 (PMC8131868; doi:10.3389/fcimb.2021.672472)

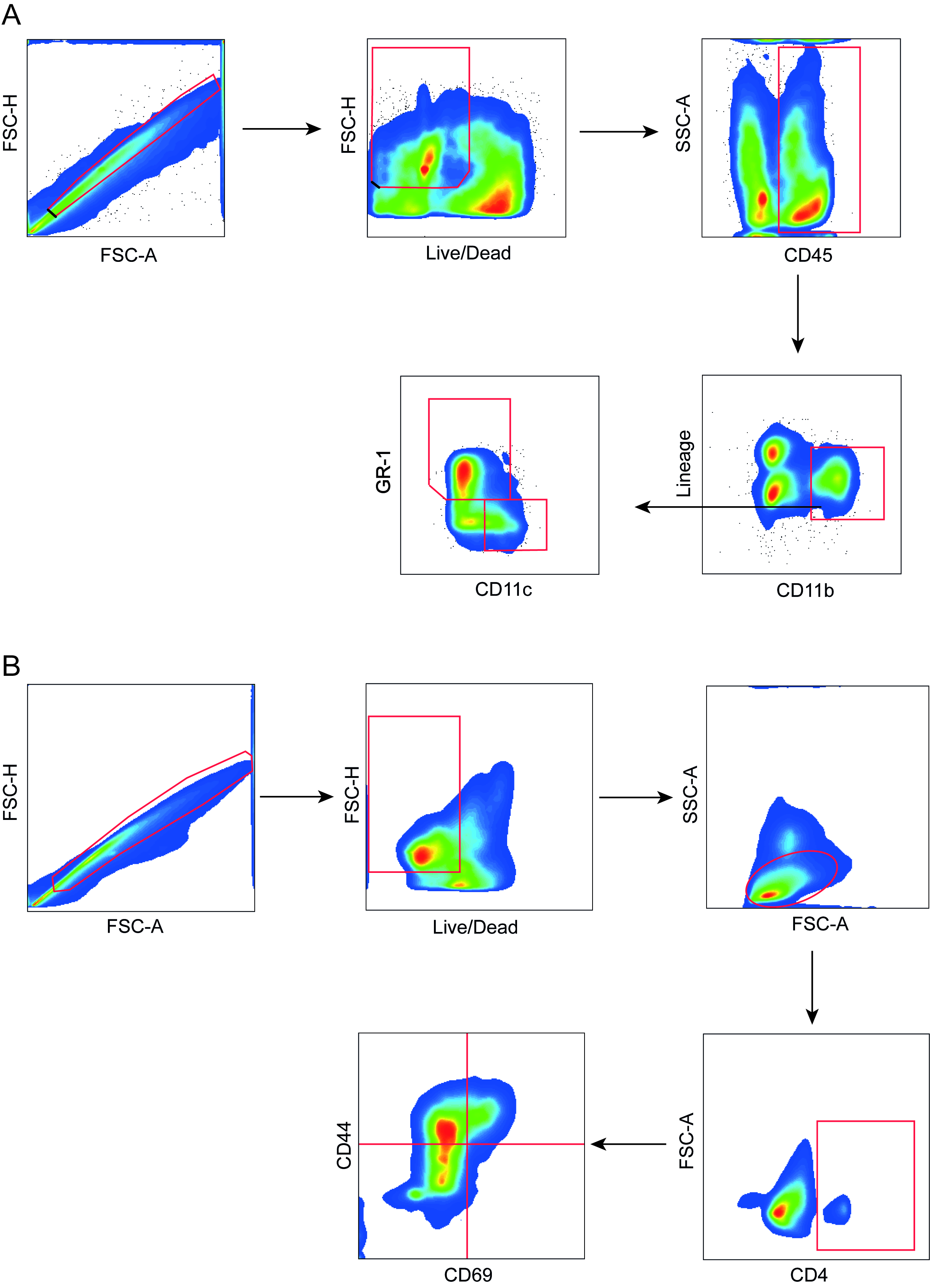

Supplement: Supplementary Figure 1 — Gate strategies for the analyses of myeloid cells and CD4+ T cells are shown in mice infected with MP287/03 mycobacteria, treated or not treated with BBG. C57BL/6 mice were infected i.t. with ~100 MP287/03 bacilli. Lung cells were evaluated at day 28 of infection. (A) Gate strategies to evaluate CD45+, CD45+Lin-CD11b+, CD45+Lin-CD11b+CD11c+, CD45+Lin-CD11b+GR1+ cells are shown. (B) Gate strategies to evaluate CD4+ T cells and CD69+CD44+CD4+ T cells are shown. [file Image_1.tif]

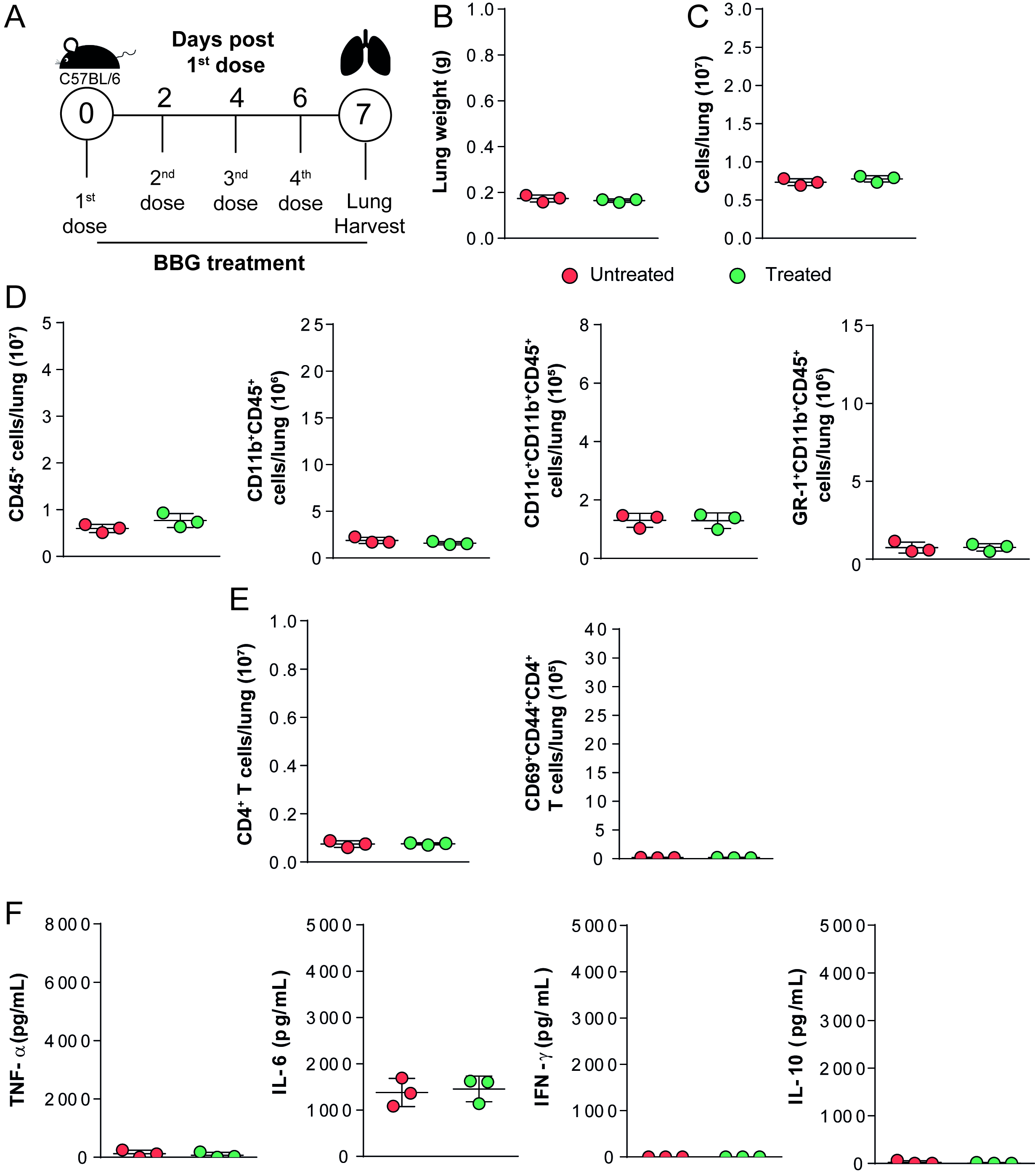

Supplement: Supplementary Figure 2 — BBG treatment does not interfere with pulmonary immunological parameters in uninfected C57BL/6 mice. Four doses of BBG (45 mg/kg) were administered i.p. every two days in C57BL/6 mice, and lung cells were harvested 7 days after the first dose. Untreated C57BL/6 mice were used as controls. (A) Schematic representation of the experimental BBG treatment protocol is shown. (B) Lung weights are shown. (C) Cell numbers per lung are shown. (D) CD45+, CD11b+CD45+ and CD11c+CD11b+CD45+, GR1+CD11b+CD45+ cell numbers per lung are shown. (E) CD4+ and CD69+CD44+CD4+ T cell numbers per lung are shown. (F) Cytokine levels in 48h-culture supernatants of lung cells are shown. No significant difference was observed using Mann-Whitney non-parametric T test. [file Image_2.tif]
